# Genome Sequence of a Novel HIV-1 Circulating Recombinant Form (CRF77_cpx) Identified among Blood Donors in Malaysia

**DOI:** 10.1128/genomeA.00459-17

**Published:** 2017-06-29

**Authors:** Kok Keng Tee, Abdul Hamid Bon, Wei Zhen Chow, Kim Tien Ng, Kok Gan Chan, Adeeba Kamarulzaman, Michael P. Busch, Yutaka Takebe

**Affiliations:** a Department of Medical Microbiology, Faculty of Medicine, University of Malaya, Kuala Lumpur, Malaysia; b Centre of Excellence for Research in AIDS (CERiA), Faculty of Medicine, University of Malaya, Kuala Lumpur, Malaysia; c National Blood Centre of Kuala Lumpur (NBCKL), Kuala Lumpur, Malaysia; d Division of Genetics and Molecular Biology, Institute of Biological Sciences, Faculty of Science, University of Malaya, Kuala Lumpur, Malaysia; e Blood Systems Research Institute (BSRI), San Francisco, California, USA; f Department of Laboratory Medicine, University of California, San Francisco, San Francisco, California, USA; g AIDS Research Center, National Institute of Infectious Diseases, Toyama, Tokyo, Japan

## Abstract

We report here the first HIV-1 circulating recombinant form (CRF) complex identified among the blood donors in Malaysia. The CRF77_cpx mosaic genome consists of parental subtypes B′, C, and CRF01_AE and is structurally related to CRF07_BC. The identification of CRF77_cpx underlines the genetic complexity and mobility of HIV-1 among the blood donors.

## GENOME ANNOUNCEMENT

The prevalence of HIV-1 infection among blood donors in Malaysia has been reported at 0.04%, presenting a major risk for unsafe blood transfusion due to the window of acute infection. A recent study showed an extensive molecular diversity of HIV-1 among the infected blood donors in the country caused by the increased spread of the recombinant HIV-1 lineages, known as the circulating recombinant forms (CRFs), and the cross-border migration of previously unreported genotypes from highly prevalent countries (1). Here, we describe the genome sequence of a novel HIV-1 recombinant complex, designated CRF77_cpx by the Los Alamos National Laboratory, detected among blood donors in Kuala Lumpur, Malaysia, in 2013 and 2014.

HIV-1 RNA was extracted from four consenting subjects (13MYNBB108, 14MYNBB084, 14MYNBB090, and 14MYNBB164) and amplified using sets of primers that spanned the complete HIV-1 genome (2), including the *gag*, *pol*, *env*, *tat*, *rev*, *vif*, *vpr*, *vpu*, and *nef* genes and the noncoding 5′- and 3′-long terminal repeats. The contiguous nucleotide sequences generated by the 3730xl DNA Analyzer (Applied Biosystems, USA) were assembled and codon aligned with the reference sequences retrieved from the HIV database (http://www.hiv.lanl.gov). The genomes were analyzed for the presence of recombinant structures using the Recombinant Identification Program (RIP) in the HIV database. To determine the precise recombination structures of these genomes, the closely related putative parental genotypes, namely, subtype B' (93CNRL42), subtype C (95IN21068), and CRF01_AE (90THCM235), determined by similarity plot were used for bootscan and informative site analyses (3). Subgenomic phylogenetic trees were reconstructed to confirm the clustering of each region. The study was approved by the University of Malaya Medical Centre (UMMC) medical ethics committee.

All CRF77_cpx genomes formed a monophyletic cluster distantly related to all known HIV-1 subtypes and CRFs with distinct recombination structure displayed in the RIP analysis. Comparative analysis showed 10 identical recombination breakpoints located in the *gag* (6 breakpoints), *pol* (3 breakpoints), and *env* genes (1 breakpoint), forming 11 genomic regions of different ancestral origins: region I (relative to HXB2 numbering system, nucleotides [nt] 708 to 1197), region II (nt 1245 to 1356), region III (nt 1380 to 1557), region IV (nt 1563 to 1636), region V (nt 1691 to 1962), region VI (nt 1971 to 2100), region VII (nt 2133 to 2987), region VIII (nt 3032 to 3164), region IX (nt 3206 to 4739), region X (nt 4988 to 8765), and region XI (nt 8784 to 9569). The origin of each region was traced to the respective parental lineages by maximum likelihood phylogenetic analysis; regions I, III, V, VII, IX, and XI were grouped with subtype C, regions II, IV, VI, and VIII were grouped with subtype B', and region X was grouped with CRF01_AE. Interestingly, regions I to IV and VIII of CRF77_cpx shared identical recombination structure with CRF07_BC, one of the oldest CRFs that was first discovered in China in the 1990s (4). CRF07_BC is highly prevalent in mainland China and Taiwan (5, 6), while its circulation in the Southeast Asia region has rarely been reported. The identification of a novel HIV-1 CRF77_cpx in Malaysia that was linked to lineages from highly prevalent countries highlights the genetic complexity and mobility of HIV-1 lineages among the blood donors.

### Accession number(s).

The genome sequence of HIV-1 CRF77_cpx isolates 13MYNBB108, 14MYNBB084, 14MYNBB090, and 14MYNBB164 are available in GenBank under accession numbers KX673818 to KX673821, respectively.
